# Association between urban environment and mental health in Brussels, Belgium

**DOI:** 10.1186/s12889-021-10557-7

**Published:** 2021-04-01

**Authors:** Ingrid Pelgrims, Brecht Devleesschauwer, Madeleine Guyot, Hans Keune, Tim S. Nawrot, Roy Remmen, Nelly D. Saenen, Sonia Trabelsi, Isabelle Thomas, Raf Aerts, Eva M. De Clercq

**Affiliations:** 1 Risk and Health Impact Assessment, Sciensano, Rue Juliette Wytsman 14, BE-1050 Brussels, Belgium; 2 Applied Mathematics, Computer Science and Statistics, Ghent University, Krijgslaan 281, S9, BE-9000 Ghent, Belgium; 3 Epidemiology and Public Health, Sciensano, Rue Juliette Wytsman 14, BE-1050 Brussels, Belgium; 4 Department of Veterinary Public Health and Food Safety, Ghent University, Salisburylaan 133, Hoogbouw, BE-9820 Merelbeke, Belgium; 5 Louvain Institute of Data Analysis and Modelling in Economics and Statistics, UCLouvain, Voie du Roman Pays, 34 bte L1.03.01, BE-1348 Louvain-La-Neuve, Belgium; 6 Nature and Society, Own-Capital Research Institute for Nature and Forest (EV-INBO), Vlaams Administratief Centrum Herman Teirlinckgebouw, Havenlaan 88 bus 73, BE-1000 Brussels, Belgium; 7 Centre of General Practice, University of Antwerp, Doornstraat 331, BE-2610 Antwerp, Belgium; 8 Center for Environmental Sciences, University of Hasselt, Agoralaan D, BE-3590 Hasselt, Belgium; 9 Center for Environment and Sciences, Department of Public Health and Primary Care, University of Leuven (KU Leuven), Herestraat 49-706, BE-3000 Leuven, Belgium; 10 Fund of scientific research, FNRS, Brussels, Belgium; 11 Division Ecology, Evolution and Biodiversity Conservation, University of Leuven (KU Leuven), Kasteelpark Arenberg 31-2435, BE-3001 Leuven, Belgium

**Keywords:** Environmental epidemiology, Air pollution, Green space, Noise, Building morphology, Mental health

## Abstract

**Background:**

Mental health disorders appear as a growing problem in urban areas. While common mental health disorders are generally linked to demographic and socioeconomic factors, little is known about the interaction with the urban environment. With growing urbanization, more and more people are exposed to environmental stressors potentially contributing to increased stress and impairing mental health. It is therefore important to identify features of the urban environment that affect the mental health of city dwellers. The aim of this study was to define associations of combined long-term exposure to air pollution, noise, surrounding green at different scales, and building morphology with several dimensions of mental health in Brussels.

**Methods:**

Research focuses on the inhabitants of the Brussels Capital Region older than 15 years. The epidemiological study was carried out based on the linkage of data from the national health interview surveys (2008 and 2013) and specifically developed indicators describing each participant’s surroundings in terms of air quality, noise, surrounding green, and building morphology. These data are based on the geographical coordinates of the participant’s residence and processed using Geographical Information Systems (GIS). Mental health status was approached through several validated indicators: the *Symptom Checklist-90-R* subscales for depressive, anxiety and sleeping disorders and the *12-Item General Health Questionnaire* for general well-being. For each mental health outcome, single and multi-exposure models were performed through multivariate logistic regressions.

**Results:**

Our results suggest that traffic-related air pollution (black carbon, NO_2_, PM_10_) exposure was positively associated with higher odds of depressive disorders. No association between green surrounding, noise, building morphology and mental health could be demonstrated.

**Conclusions:**

These findings have important implications because most of the Brussel’s population resides in areas where particulate matters concentrations are above the World Health Organization guidelines. This suggests that policies aiming to reduce traffic related-air pollution could also reduce the burden of depressive disorders in Brussels.

**Supplementary Information:**

The online version contains supplementary material available at 10.1186/s12889-021-10557-7.

## Background

Mental health disorders appear to be a growing problem in modern societies, specifically in urban areas [1]. According to the World Health Organization (WHO), depression affects around 264 million people and is one of the main causes of disability worldwide [2]. Nearly the same amount of people suffer from anxiety disorders and many people experience both conditions simultaneously [3]. While mental health disorders are generally linked to demographic and socioeconomic factors, little quantitative data is available on the interaction between mental health and the urban environment. With growing urbanization, more and more people are exposed to environmental stressors, potentially contributing to increased stress and impairing mental health [4, 5]. It is therefore important to identify specific features of the urban environment that might affect the health of the city dwellers. When studying the impact of the urban environment on mental health, the main focus goes to air pollution, urban greenness, noise and urban morphology.

Air pollution, largely attributed to traffic volume, has been associated with mental disorders in several studies [6–12]. In the light of their toxicity on the central nervous system, air pollutants may have a possible role in the onset or worsening of mental conditions [13, 14]. Air pollution exposure may lead to oxidative stress, neuro-inflammation, cerebrovascular damage, and neurodegenerative pathology [15]. Air pollution has also been associated with behavioral determinants of mental health such as spending less time outdoors, reduced physical activity [16, 17] and contact with nature, limited exposure to sunlight and vitamin D deficiency [18]. Among the common indices of air pollution, fine particulate matter (*PM*_*2.5*_) appears to play an influential role on depression and on psychotic disorders [19, 20]. Also, nitrogen oxides (*NO*_*x*_), particularly nitrogen dioxide (*NO*_*2*_), seem to have a significant place among the risks factors of psychotic disorders. Regarding ambient ozone exposure (*O*_*3*_), current evidence for an association with mental health remains inconclusive [21]. While there has been an increasing number of studies investigating the association between PM and mental health, few studies have examined the potential impact of black carbon (*BC*). According to toxicological studies, *BC* may operate as an universal carrier of a large variety of toxic chemicals in the human body and could be a more suitable air quality indicator to evaluate the health risks of traffic-related air pollution [22].

A green environment has been associated with a reduced risk of poor mental health in several reviews and epidemiological studies [23–28]. Different theories were suggested based on three benefits attributed to green spaces: (i) reducing exposure to environmental stressors such as noise, heat, and air pollution, (ii) facilitating social cohesion and physical activities, and (iii) reducing stress levels [29].

The mechanism for stress reduction requires visual perception of green space. However, measures of green exposure are commonly assessed at the neighbourhood level and do not capture street-level exposures [30]. Green exposure is often assessed through a “standard” set of measures (i.e. greenness, quantified by the Normalized Difference Vegetation Index) that does not incorporate information on specific features of urban greenness that might drive health outcomes and present a risk of oversimplifying the perceptions of urban dwellers of their environment [31, 32]. For instance, it has been shown that tree density, assessed on the ground and not by remote sensing, had a significant influence on reducing oppressiveness [33, 34].

Noise is another prominent feature within the urban environment. Recent reviews have revealed that transportation noise such as road, aircraft or rail traffic noise leads to sleep disturbance [35]. Increasing exposure to road traffic noise has also been associated with depression and anxiety in recent systematic reviews and meta-analyses, yet quality of evidence was considered as “very low” [36, 37]. However, poor quality of evidence does not mean that noise should not be considered as a risk factor for mental disorders [35], as the relationship between noise and mental health is biologically plausible [38]. Several studies support the hypothesis that noise is associated with neurocognitive functions, mood disorders and neurodegenerative disease [38, 39].

The urban building morphology may also have a potential impact on mental health [40]. The street canyon effect, where narrow streets are flanked by high buildings on both sides, may reduce light penetration and increase noise volume at street level [41, 42]. Also the shape of high-rise buildings may have an oppressive impact on dwellers [43]. Urban canyons contribute to the urban heat island effect and poor air quality by reducing the capacity for pollutants released by traffic to dissipate [44, 45]. All this may impact the mental health of city dwellers. The street corridor effect is another common characteristic of the building structure of urban streets and is determined by the ratio between the distance between parallel facades and street length. To our knowledge, no study has yet assessed the potential impact of the street canyon and corridor effect on mental health.

The exposures to air pollution, surrounding green, noise and characteristics of the built environment are generally spatially correlated [29]. However, most of the epidemiological studies assessing the relation between the built environment and mental health are single-exposure models [5, 32]. Evaluating these environmental exposures separately ignores the potential confounding effects between them [5, 32]. It remains thus unclear to what extent associations between urban greenness and mental health are attributable to air pollution, and vice versa. Lifestyle factors, such as physical activity and social support can also mediate or confound the relation between environment and mental health [46]. Another shortcoming of existing studies is that mental health is often approached by a single indicator making it difficult to grasp the different dimensions of mental health and to compare results across studies [4, 32].

The aim of this study is to define associations of combined long-term exposure to air pollution, surrounding green at different scales, noise from multiple sources, and urban building morphology with several dimensions of mental health in Brussels. This research is part of a wider project called Nature Impact on Mental Health Distribution (NAMED) which aims to generate a comprehensive understanding of associations between mental health and the urban residential environment [47].

## Methods

### Study *area*

Brussels is the capital city of Belgium. Whereas the larger delineation of the urban agglomeration is much discussed [48], the highly urbanized city-center constitutes an administrative Region named the Brussels-Capital Region (here noted BCR). It is divided into 19 municipalities.

The BCR counts 1,198,726 inhabitants (01/01/2018) and is 161 km^2^ large [Source: Statistics Belgium]. The population of the BCR is characterized by a high cultural diversity: one in three inhabitants does not have the Belgian nationality and one in two was not born in Belgium [49]. In Belgium, the psycho-emotional health of the population has deteriorated in recent years, more specifically in the BCR compared to the other two regions, Wallonia and Flanders [50].

In comparison to other European agglomerations with more than 100,000 inhabitants, the BCR offers a high percentage of urban green [51]. However, green space is unequally distributed with the largest urban green coverage situated in the south-east of the BCR [52].

### Study *population* and data

Data was extracted from the Belgian Health Interview Surveys (HIS) conducted in 2008 and 2013. The HIS is a national cross-sectional epidemiological survey carried out every five years by Sciensano, the Belgian Institute for Health, in partnership with Statbel, the Belgian statistical office. In order to ensure the representativeness of the Belgian population, a stratified multistage, clustered sampling of the population was applied. The surveys cover are socio-economic status (SES), physical and mental health, and lifestyle [53].

Only participants older than 15 years, living at the same place of residence in the BCR for at least one year and who completed the entire set of questions, were included in the sample (*n* = 1325). The dataset was further enriched with objective measures of the residential urban environment, based on the geographical coordinates of the residential address of participants and processed using Geographical Information Systems (GIS). (see Section 2.4).

### Indicators of mental health

The mental health status of HIS participants was approached through different validated tools:

The General Health Questionnaire (GHQ-12) [54] for general well-being is a commonly used screening tool that detects symptoms consistent with poor mental health during the last 2 weeks. The format is a 12-item test with a four-point scale for each response. It includes questions such as: “In the last 2 weeks, were you: (i) able to concentrate, (ii) capable of making decisions, (iii) under stress etc.” with the following possible answers: “as usual”, “better than usual”, “less than usual”, “much better than usual”.

Using the standard bimodal scoring method (0–0–1-1), the GHQ-12 yields a crude score ranging from 0 to 12 where a cut-off at 4 defines people with probable mental health disorders. The GHQ-12 permits to identify temporary alterations of normal psychological functioning and is sensitive to common psychological disorders, like depression and anxiety [55]. We used the dichotomized indicator *GHQ-4* with the cut-off value at 4. This cut-off is used in the health interview surveys of other countries and allows international comparisons.

The Symptom Checklist-90-R (hereafter SCL-90R) is a validated tool designed to evaluate a broad range of psychological problems and symptoms of psychopathology. The questionnaire includes 42 items distributed in subscales corresponding to symptoms of different disorders, with a five-point scale for each response. It includes questions such as: “In the last week, to what extent did you feel the following difficulties: (i) feeling no interest in things, (ii) feeling low in energy, (iii) crying easily etc.” with the following possible answers: “Not at all”, “a little bit”, “moderately”, “quite a bit”, “extremely”.

We used the subscales for depressive (13 items), anxiety (10 items) and sleeping disorders (3 items). Three binary indicators were calculated on basis of responses to the SCL-90R subscales: the likelihood of presenting *anxiety disorders*, *depressive disorders* and *sleeping disorders*. Subscale scores were calculated as the sum of the items (with 5 options: 0, 1, 2, 3, and 4) divided by the number of items of the subscale. The obtained scores were dichotomized with a cut off at 2 (i.e., [0–1] versus [2–4]).

Not all questions were answered by all participants in the study, which resulted in missing data for a number of questions. Lines with missing data were removed from further analysis. No imputation for missing data was performed since the reason for non-responding was unknown.

## Objective measures of the environment

### Air pollution

**Fig. 1:**
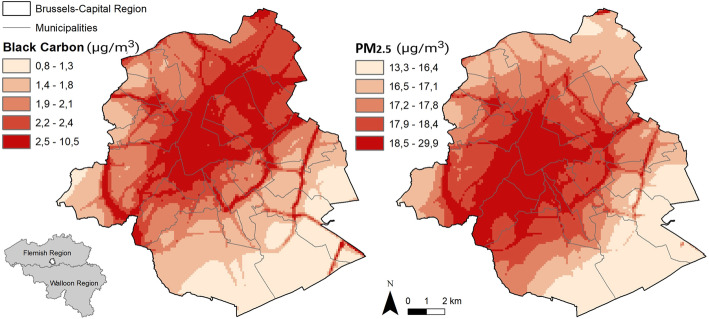
Air pollution exposure in Brussels. Annual mean (2013) of BC and PM_2.5._ Data source: IRCEL - CELINE

Exposure at the residence address of participants was obtained through the national monitoring system supervised by the Belgian Interregional Environment Agency (IRCEL – CELINE). Concentrations of various pollutants are assessed on a daily basis through a dense network of stations distributed all over the country. The measurements are interpolated to estimate local exposure taking into account land cover data in combination with a dispersion model [56–58]. The accuracy of the model to evaluate an individual’s real exposure has already been shown in a study comparing modelled particulate matter (*PM*_*2.5*_) and black carbon (*BC*) at the residence with internal exposure measured in urine [59]. Annual average in the year of HIS participation of *PM*_*2.5*_*, PM*_*10*_*, BC*, ozone (*O*_*3*_) and nitrogen dioxide (*NO*_*2*_) at the participant’s residence address were used as indicators of air quality. All the air pollution indicators were used as continuous and categorical variables (tertiles) (Fig. 1).

### Urban greenness

Three sources were used to measure urban greenness: (i) the Google Street View panorama, (ii) urban tree data from the UrbIS open database 2018 [60] and (iii) vegetation coverage data, based on high-resolution remote sensing data (Normalized Difference Vegetation Index threshold value of 0.275) provided by Brussels Environment, the local environment and energy administration (computed by Van de Voorde et al.) [61]. NDVI was not used as such but transformed into a binary variable (presence/absence of vegetation).

Urban greenness was assessed at three different levels:

At the residence level, the computation of the *view of green* is based on the closest Google Street View panorama to the residential address based on the method of Li et al [62]. The *view of green* is the ratio of the total green area from Google Street View panorama (~picture 360°) to the total area of the panorama. Unfortunately, the season of the pictures could not be controlled for. This measure provides a green view indicator with a ground-level perspective and captures the eye-level street greenery from the doorstep. The variable was used as a continuous and categorical variable (tertiles).

At the street level (residential street segment, delimited by two intersections), the *linear tree density* and the *visible street vegetation coverage* indicators were computed. *The linear tree density* is the ratio between the number of trees (point data from Urbis) and the street length. Alignments of trees were taken independently because they are not always detected by remote sensing. The *visible street vegetation coverage* indicator is the vegetation coverage (from Brussels Environment) on the street, and 10 m on either side. This takes into account the vegetation that is visible from the street such as front gardens, trees in the street and small green spaces. The two indicators were used as continuous and categorical variables. *The visible street vegetation coverage* was categorized in tertiles. Because of the high proportion of zero values, the *linear tree density* was categorized in this way: no trees, < median, > median of the remaining values.

**Fig. 2:**
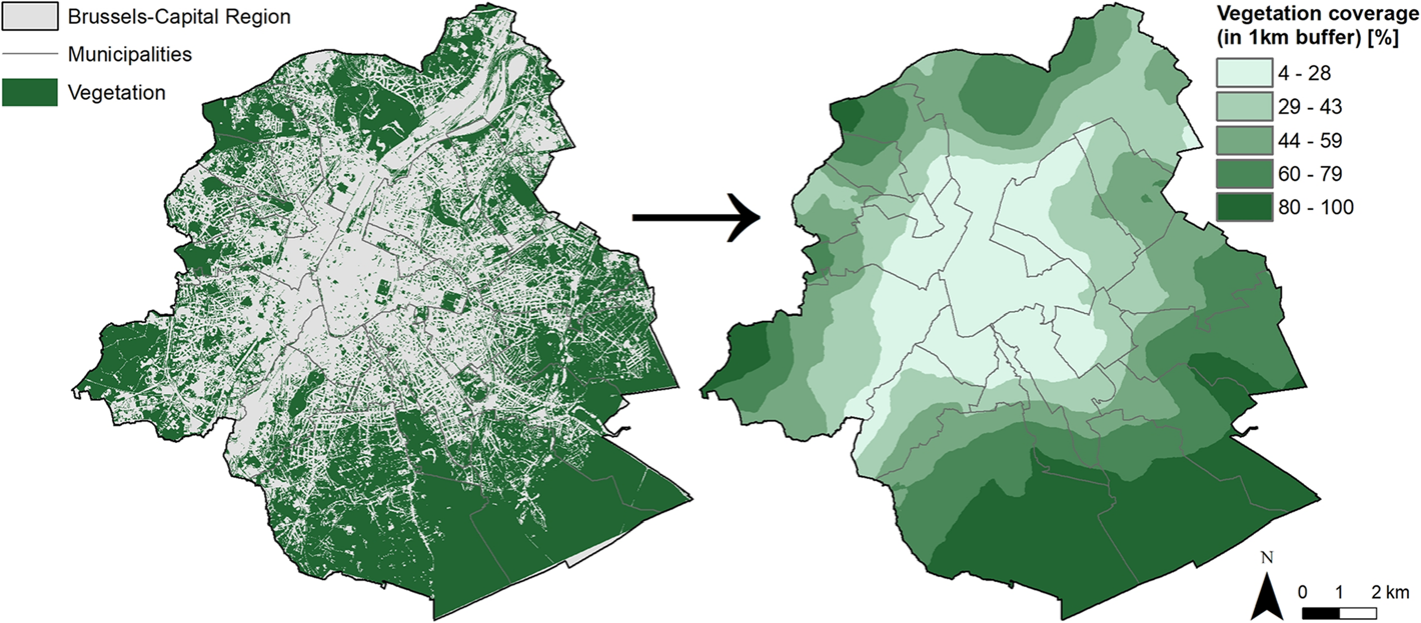
Vegetation distribution in Brussels. Vegetation distribution in Brussels (left) used to compute a green cover indicator (right, here for a 1 km buffer). Data source: Van de Voorde et al., 2010 and Urbis database, 2017

At the neighborhood level, vegetation coverage was assessed within two different buffers (600 and 1000 m). This more traditional indicator of vegetation was calculated by taking the ratio of vegetation coverage (from Brussels Environment) in a 600 m (or 1000 m) circle around the respondent’s dwelling (Fig. 2). The two indicators were used as continuous and categorical variables (tertiles).

### Noise

An already developed GIS-based noise model was available to estimate residential noise levels as required by the European Noise Directive (2002/49/EC) [63, 64]. The noise database maps noise from respectively road, rail and air traffic and allows an assessment of population exposure across Europe according to harmonized indicators such as day–evening–night noise level (Lden) [65]. The Lden indicator is an average sound pressure level over all days (12 h), evenings (4 h) and nights (8 h) in a year. Noise maps available for Brussels were obtained from Bruxelles Environnement for the years 2006 and 2011 [66, 67] and were combined with the geographical coordinates of the participants’ residence to estimate Lden noise values in 5 dB(A) intervals. Noise pollution was approached through the *noise from multiple sources* (Lden) indicator since this included most information on residential noise.

### Urban building morphology

**Fig. 3:**
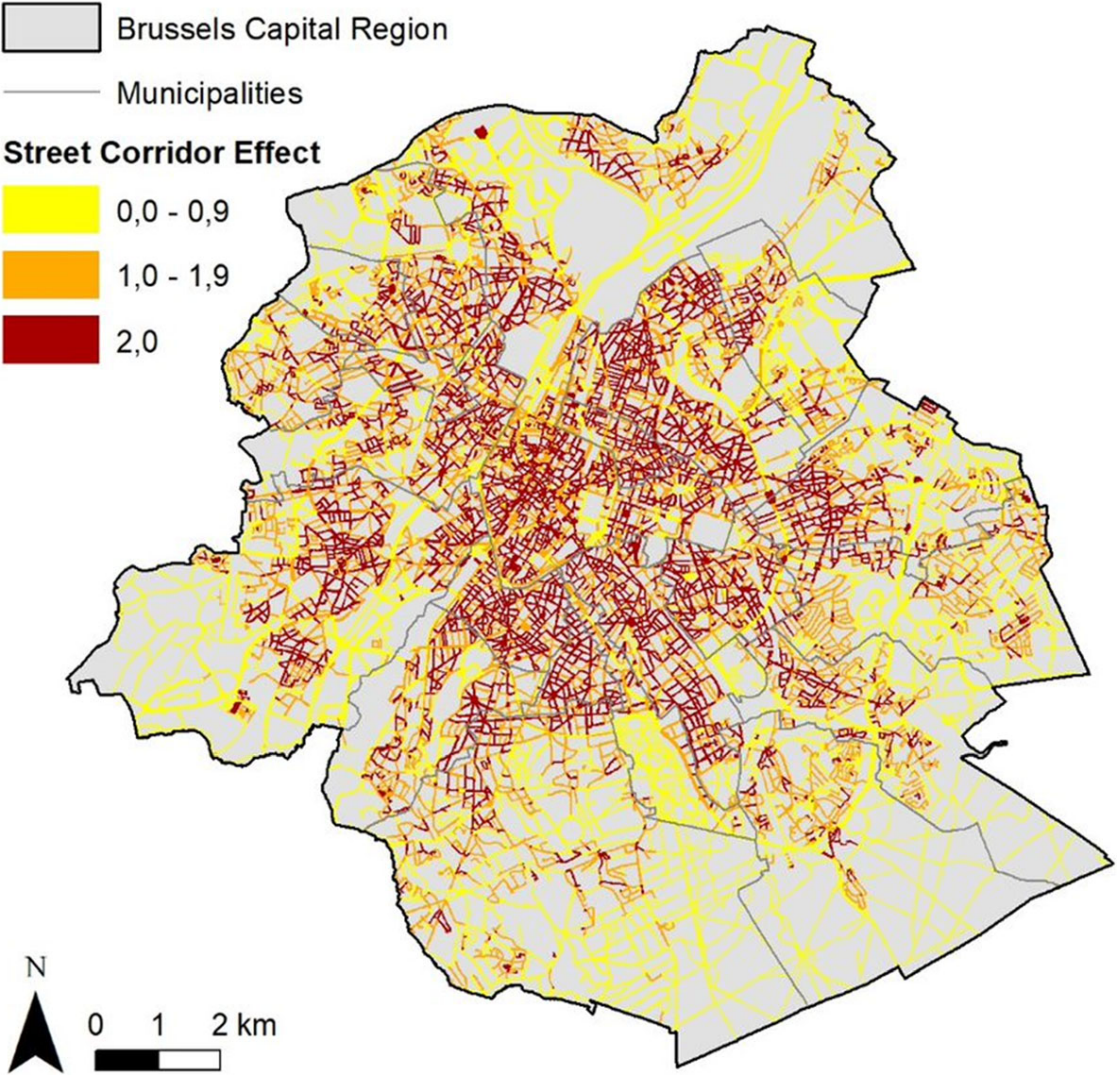
*Street corridor effect* in Brussels (ratio between parallel facades length and street length). Data source: Urbis data 2017

Two indicators were developed to assess the building morphology at the street level: the *street corridor effect* (Fig. 3) and the *street canyon effect.* The *street canyon effect* or height/width ratio is the ratio between average building height and average open space width, while the *street corridor effect* is the ratio between parallel facades length and street length. This was computed using the Urbis database [60]. The two indicators were treated as continuous and categorical variables in the analyses. The *street canyon effect* was categorized in tertiles. Regarding the *street corridor effect*, because half of the participant’s live in a street with a maximum *street corridor effect* (ratio = 2), the variable was categorized in this way: maximum corridor effect [2], < median, > median of the remaining values.

## Indicators of socio-economic status and lifestyle

To describe participant’s socio-economic status, we used: *age*, *sex*, *household composition*, *highest educational level in the household* and the *reported household income*. The *highest educational level in the household* was categorized in 3 groups: no diploma or primary education or lower secondary, higher secondary, higher. Other covariates related to lifestyle were included in the multi-exposure models: the *level of physical activity* based on the short version of the International Physical Activity Questionnaire (IPAQ), the *perceived quality of social support* (poor versus intermediate or strong support) and the *chronic condition* (from the Minimum European Health Module) [68]. For the *level of physical activity*, the proposed levels are: (i) inactive (no activity is reported or some activity is reported but not enough to meet categories 2 or 3) (ii) minimally active (3 or more days of vigorous activity of at least 20 min per day or 5 or more days of moderate activity or walking of at least 30 min per day or 5 or more days of any combination of walking, moderate or vigorous activities achieving a minimum of at least 600 MET-min/week) (iii) health-enhancing physical activity (vigorous activity on at least 3 days and accumulating at least 1500 METminutes/week OR 7 or more days of any combination of walking, moderate or vigorous activities achieving a minimum of at least 3000 MET-minutes/week).

### Statistical analysis

All mental health, environmental and socio-economic indicators were described with their 95% confidence interval. The continuous variables were described by their median and the 25th and 75th percentile.

The selection of the environmental indicators included in the multi-exposure regression models was based on the results of previous work, where the relationship between all continuous environmental indicators has been explored through a Principal Component Analysis (PCA) [69].

Only *BC* was included in the multi-exposure models for the following main reasons: (i) all the air pollutants were strongly associated and could therefore not all be included in the model (problem related to the variance inflation factor [VIF]), (ii) it has been shown that *BC* represents one of the most health-relevant components of PM and could be a valuable indicator to assess the health effects of air quality dominated by primary combustion particles. According to toxicological studies, *BC* may operate as universal carrier of a large variety of toxic chemicals to the human body and could be a more suitable air quality indicator to evaluate the health risks of traffic-related air pollution.

For each regression model the VIF was used to quantify multi-collinearity between the explanatory variables. A VIF value above 5 was used as a threshold.

Correct estimates and valid inferences were obtained by taking the survey weights, strata and clusters relative to the sample design into account. All analyses were performed using the statistical software STATA 14 using the SVY option.

### Single exposure models

For each mental health outcome, single-exposure models were fitted using multivariable logistic regressions. Models were adjusted for *age*, *sex*, *family composition*, *reported household income*, *highest educational level in the household* and *year*. Results were reported for both tertiles and continuous terms of the environmental variables. These models were developed for each of the 13 considered environmental stressors: *BC, PM*_*2.5*_*, PM*_*10*_*, NO*_*2*_*, O*_*3*_*, View of green, Street visible vegetation coverage, linear tree density, vegetation coverage 600 m, vegetation coverage 1000 m, noise from multiple source (Lden), street corridor effect, street canyon effect.*

### Multi-exposure models

For each mental health outcome, multi-exposure models were fitted using multivariable logistic regressions with increasing adjustment for covariates. Model 1 included only the exposure variables without adjustment for socio-economic factors. Model 2 was adjusted for socio-economic factors. Model 3 was additionally adjusted for lifestyle factors, such as physical activity and perception of social support. Model 4 was additionally adjusted for chronic conditions.

All variables were included as continuous variables. In order to capture the potential non-linear association between the environmental factors and each metal health outcome, we included a quadratic term of each environmental factor in each model. To avoid problem of multicollinearity between the environmental factor and his quadratic term, variables were normalized by subtracting their mean. The quadratic term was retained in the model when it significantly improved the model, according to the Wald test. Interactions were tested between each of the environmental factors and age and sex.

- ***Model 1:*** *Mental health ~ BC + view of green Index + linear tree density + vegetation coverage 1 km buffer + noise from multiple source (Lden) + street corridor effect + street canyon effect.*
- ***Model 2:*** *Mental health ~ BC + view of green Index + linear tree density + vegetation coverage 1 km buffer + noise from multiple source (Lden) + street corridor effect + street canyon effect + SES.*
- ***Model 3:*** *Mental health ~ BC + view of green Index + linear tree density + vegetation coverage 1 km buffer + noise from multiple source (Lden) + street corridor effect + street canyon effect + SES + social support + physical activity.*
- ***Model 4:*** *Mental health ~ BC + view of green Index + linear tree density + vegetation coverage 1 km buffer + noise from multiple source (Lden) + street corridor effect + street canyon effect + SES + social support + physical activity + chronic condition.*

Due to the high number of tests performed on the same dataset, we might obtain a false positive finding. To face this problem, we used the Benjamini-Hoshberg method to control for multiple hypotheses testing.

### Mediation analysis

We used structural equation models (SEMs) to analyze the multivariate relationships in our dataset. We constructed two a-priori models of associations between physical activity, poor social support, year, noise and the latent variables “distress”, “green space”, “socio-economic status” (SES), and “air pollution”. Distress was estimated from the binary variables *depressive disorders*, *anxiety disorders*, *sleeping disorders* and *GHQ ≥ 4*. The four indicators of distress had high internal consistency (Cronbach’s alpha = 0.73).

Green space was estimated from *linear tree density*, *view of green*, *vegetation coverage within 600 m* distance of the residence, *vegetation coverage within 1000 m* of the residence and *street visible vegetation coverage*. Socio-economic status was estimated from the *highest educational level in the household* and the *reported household income*. Noise was estimated from mean annual exposition to *noise from multiple source* (air traffic, rail traffic, and road traffic) Lden. Air pollution was estimated from mean annual concentrations of *PM*_*10*_, *PM*_*2.5*_, *NO*_*2*_, *BC*, and *O*_*3*_. All continuous variables were first transformed in z-scores, by subtracting the mean and dividing by the standard deviation. In a first SEM, we hypothesized that green space would lead to reduced air pollution, increased physical activity and better social support; and that distress would be associated with lower amounts of green space, lack of physical activity, poor social support, low SES, air pollution and noise. The second SEM differed from the first in two ways: we hypothesized that green space would be associated with lower levels of air pollution only, and that air pollution would affect physical activity and social support. We always included a direct effect of year on distress to account for unmeasured variables that may have an impact on distress and that differed between the two survey years. The *p*-value of the chi-square statistic, the root mean square error of approximation (RMSEA), the standardized root mean square residual (SRMR) and the comparative fit index (CFI) were reported as model fit indices. We report standardized coefficients and their *p*-value. All models were fit and evaluated using the package lavaan 0.6–7 in R version 3.6.3 [70].

## Results

### Data description

A total of 1325 residents of BCR were included in the study population. Table 1 describes all considered variables.

**Table 1:** Description of the sample population

Socio-economic status	
**Age** median [IQR]/N	47 [34–63]/1325
**Sex** % [95% CI]/N	
M	50.59 [47.70–53.47]/616
F	49.41 [46.53–52.30]/709
**Year** % [95% CI]/N	
2008	53.93 [50.19–57.62]/775
2013	46.07 [42.38–49.81]/550
**Highest educational level in the household** % [95% CI]/N	
No diploma/primary education/lower secondary	19.34 [16.75–22.21]/285
High secondary	26.77 [23.67–30.12]/365
Higher	53.89 [50.22–57.52]/675
**Household composition** % [95% CI]/N	
Single	35.10 [31.70–38.67]/416
One parent with child [ren]	10.92 [8.91–13.32]/145
Couple without child [ren]	18.86 [16.26–21.76]/277
Couple with child [ren]	29.03 [25.64–32.66]/395
Other/unknown	6.10 [4.53–8.15]/92
**Reported household income** median [IQR]/N	1460 [1000–2000]/1325
**Mental health status**	
**Probable mental disorders [GHQ ≥ 4]** % [95% CI]/N	19.81 [17.40–22.46]/255
**Anxiety disorders** % [95% CI]/N	9.36 [7.74–11.29]/124
**Depressive disorders** % [95% CI]/N	15.79 [13.61–18.25]/216
**Sleeping disorders** % [95% CI]/N	28.22 [25.19–31.46]/351
**Environmental factors**	
**Black carbon [annual mean μg/m**^**3**^**]** median [IQR]	2.24 [1.95–2.62]/1325
**NO**_**2**_ **[μg/m**^**3**^**]** median [IQR]	34.64 [32.04–37.70]/1325
**PM**_**2.5**_ **[μg/m**^**3**^**]** median [IQR]	19.16 [17.82–20.11]/1325
**PM**_**10**_ **[μg/m**^**3**^**]** median [IQR]	26.14 [24.11–26.53]/1325
**O**_**3**_ **[μg/m**^**3**^**]** median [IQR]	36.25 [34.49–37.73]/1325
**Vegetation coverage 600 m buffer [%]** median [IQR]	35.95 [21.64–51.48]/1325
**Vegetation coverage 1 km buffer [%]** median [IQR]	37.96 [23.80–52.5]/1325
**Street visible vegetation coverage [%]** median [IQR]	3.99 [0.00–28.38]/1325
**View of green** median [IQR]	11.28 [6.04–22.36]/1325
**Linear tree density** median [IQR]	0.04 [0.00–0.15]/ 1325
**Linear tree density [categories]** % [95% CI]/N	
no trees	28.77 [25.41–32.38]/360
< median	35.64 [32.22–39.20]/483
> median	35.6 [32.19–39.12]/484
**Noise from multiple sources Lden [dB]** median [IQR]	50.96 [48.02–54.06]/1325
**Street canyon effect** median [IQR]	0.59 [0.39–0.84]/1325
**Street corridor effect [0–2]** median [IQR]	1.99 [1.46–2.00]/1325
**Street corridor effect [categories]** % [95% CI]/N	
< median	24.80 [21.74–28.17]/336
> median	24.6 [21.64–27.76]/333
2	50.6 [46.91–54.28]/656
**Lifestyle factors**	
**Perceived quality of social support** % [95% CI]/N	
Strong/intermediate support	77.56 [74.46–80.38]/1036
Poor support	22.44 [19.62–25.54]/289
**Level of health enhancing physical activity** % [95% CI]/N	
Inactive	33.33 [30.16–36.65]/468
Minimal active	40.58 [37.44–43.79]/537
Enough active	26.10 [23.39–29.00]/320
**Chronic condition** % [95% CI]/N	33.45 [30.36–36.70]/435

*Sleeping disorders* were the most frequent reported problems (28%), followed by *depressive disorders* (16%) and *anxiety disorders* (9%). The *GHQ-4* indicator indicated that nearly 20% of the study population suffers from probable mental disorders.

Regarding the environmental variables, the median of the annual mean exposure to black carbon was 2.24 μg/m^3^. While the median of exposure to *vegetation coverage (1000 m buffer)* was 38%, the median of the *view of green* was only 11%. Furthermore, 29% of the population study lived in streets without trees. Half of the study population lived in streets with a maximum *street corridor effect* (=2), with a median of exposure equal to 1.99 (IQR: 0–2). The median of exposure to the *street canyon effect* was 0.59 (IQR: 0.04–2.89). The median of the annual mean exposure to *noise from multiple from sources* was 50.96 dB (IQR: 48.02–54.06).

A correlation matrix of all the environmental factors can be found in the additional files (Additional file 1).

With regard to lifestyle, 22% of the population perceived the quality of the *social support* as poor and 33% of the population declared to suffer from a *chronic condition*. Just over a quarter of the population was physically active enough to have a positive impact on health.

## Single-exposure models

In single-exposure models, exposure to nearly all pollutants (in tertiles) was positively and significantly associated with *depressive disorders* (Tables 2 and 3).

**Table 2:** Single-exposure models

Exposure		GHQ-4		Anxiety disorders		Depressive disorders		Sleeping disorders	
		Adj. OR [95% IC]	p	Adj. OR [95% IC]	p	Adj. OR [95% IC]	p	Adj. OR [95% IC]	p
BC (μg/m^3^)	Tertile 2 vs 1	1.36 [0.89–2.07]	0.15	1.51 [0.83–2.75]	0.17	1.60 [0.99–2.59]	0.05	1.35 [0.91–2.01]	0.13
	Tertile 3 vs 1	1.24 [0.77–1.99]	0.38	1.51 [0.83–2.74]	0.17	1.98 [1.20–3.26]	0.008	1.34 [0.89–2.01]	0.16
	1 μg/m3	1.00 [0.76–1.33]	0.95	1.07 [0.79–1.45]	0.65	1.33 [0.99–1.78]	0.05	1.12 [0.88–1.43]	0.35
NO_2_ (μg/m^3^)	Tertile 2 vs 1	1.02 [0.68–1.55]	0.92	1.66 [0.94–2.93]	0.08	1.87 [1.18–2.96]	0.007	1.07 [0.73–1.58]	0.72
	Tertile 3 vs 1	0.95 [0.62–1.45]	0.8	1.51 [0.87–2.61]	0.14	1.58 [0.99–2.51]	0.05	1.20 [0.81–1.78]	0.35
	1 μg/m3	0.99 [0.96–1.03]	0.72	1.01 [0.97–1.04]	0.65	1.02 [0.99–1.05]	0.12	1.01 [0.99–1.04]	0.32
PM_2.5_ (μg/m^3^)	Tertile 2 vs 1	1.08 [0.66–1.76]	0.77	0.74 [0.39–1.42]	0.36	0.79 [0.47–1.33]	0.38	1.01 [0.63–1.60]	0.97
	Tertile 3 vs 1	1.05 [0.55–1.99]	0.88	0.80 [0.36–1.70]	0.57	1.34 [0.71–2.51]	0.36	1.60 [0.89–2.87]	0.11
	1 μg/m3	1.01 [0.85–1.20]	0.88	1.05 [0.86–1.29]	0.62	1.12 [0.95–1.32]	0.19	1.10 [0.94–1.28]	0.23
PM_10_ (μg/m^3^)	Tertile 2 vs 1	1.19 [0.74–1.93]	0.47	1.31 [0.71–2.40]	0.38	1.93 [1.13–3.29]	0.02	1.29 [0.82–2.01]	0.27
	Tertile 3 vs 1	1.10 [0.60–2.04]	0.75	1.10 [0.50–2.41]	0.82	3.02 [1.49–6.10]	0.002	1.66 [0.94–2.93]	0.08
	1 μg/m3	1.06 [0.77–1.45]	0.71	1.06 [0.71–1.58]	0.79	1.75 [1.51–2.42]	0.002	1.29 [0.96–1.72]	0.08
O_3_ (μg/m^3^)	Tertile 2 vs 1	0.82 [0.54–1.25]	0.36	1.01 [0.60–1.68]	0.95	0.59 [0.38–0.92]	0.02	0.79 [0.54–1.15]	0.22
	Tertile 3 vs 1	1.00 [0.66–1.51]	0.99	0.78 [0.45–1.35]	0.37	0.56 [0.37–0.89]	0.01	0.72 [0.49–1.05]	0.09
	1 μg/m3	0.98 [0.92–1.05]	0.64	0.97 [0.89–1.05]	0.43	0.88 [0.55–0.95]	0.001	0.95 [0.89–1.01]	0.11
Veg. coverage 600 m buffer (%)	Tertile 2 vs 1	1.28 [0.86–1.92]	0.22	1.06 [0.63–1.78]	0.81	1.12 [0.73–1.77]	0.59	1.12 [0.77–1.64]	0.53
	Tertile 3 vs 1	1.01 [0.66–1.56]	0.96	1.02 [0.61–1.72]	0.93	0.88 [0.56–1.49]	0.59	0.93 [0.64–1.36]	0.73
	25% increase	0.98 [0.80–1.21]	0.85	1.06 [0.80–1.41]	0.65	0.94 [0.75–1.17]	0.36	0.98 [0.80–1.19]	0.85
Veg. coverage 1 km buffer (%)	Tertile 2 vs 1	1.32 [0.88–1.97]	0.17	1.14 [0.68–1.90]	0.62	1.12 [0.73–1.72]	0.59	1.09 [0.76–1.56]	0.64
	Tertile 3 vs 1	0.95 [0.62–1.47]	0.82	1.04 [0.62–1.75]	0.87	0.77 [0.49–1.20]	0.25	1.13 [0.77–1.67]	0.53
	25% increase	0.99 [0.80–1.24]	0.97	1.02 [0.76–1.36]	0.89	0.91 [0.72–1.13]	0.39	1.02 [0.84–1.23]	0.86
Street visible veg. coverage (and 10 m on either sides) (%)	Tertile 2 vs 1	0.98 [0.67–1.52]	0.92	1.00 [0.61–1.65]	0.97	1.04 [0.68–1.57]	0.86	1.24 [0.84–1.80]	0.27
	Tertile 3 vs 1	0.92 [0.63–1.42]	0.68	0.80 [0.47–1.34]	0.39	0.77 [0.50–1.21]	0.26	1.08 [0.74–1.56]	0.68
	25% increase	1.02 [0.86–1.23]	0.84	1.02 [0.81–1.31]	0.81	0.91 [0.73–1.27]	0.38	0.99 [0.83–1.18]	0.88
View of green	Tertile 2 vs 1	1.23 [0.82–1.84]	0.3	1.38 [0.82–2.33]	0.22	1.03 [0.66–1.59]	0.89	0.81 [0.56–1.18]	0.27
	Tertile 3 vs 1	1.08 [0.70–1.65]	0.73	1.42 [0.82–2.45]	0.21	1.10 [0.69–1.75]	0.68	0.90 [0.60–1.34]	0.61
	10 units increase	1.01 [0.88–1.16]	0.87	1.09 [0.91–1.31]	0.33	1.07 [0.90–1.22]	0.43	0.99 [0.85–1.22]	0.83
Tree density	<P50 tree vs 0	0.92 [0.61–1.39]	0.71	0.96 [0.57–1.62]	0.87	0.82 [0.52–1.27]	0.37	0.66 [0.45–0.99]	0.04
	>P50 tree vs 0	0.99 [0.65–1.49]	0.96	0.97 [0.56–1.69]	0.92	0.90 [0.57–1.42]	0.65	0.70 [0.47–1.05]	0.09
	25 trees/100 m increase	1.03 [0.89–1.19]	0.69	0.97 [0.80–1.66]	0.93	0.99 [0.93–1.05]	0.67	0.94 [0.62–1.40]	0.76
Noise from multiple source Lden (dB)	Tertile 2 vs 1	1.00 [0.66–1.51]	0.88	0.73 [0.44–1.32]	0.34	0.86 [0.54–1.38]	0.54	1.27 [0.84–1.93]	0.25
	Tertile 3 vs 1	0.88 [0.59–1.32]	0.43	1.01 [0.59–1.73]	0.97	1.46 [0.91–2.33]	0.11	1.39 [0.95–2.02]	0.09
	1 dB	1.00 [0.98–1.04]	0.75	1.00 [0.97–1.03]	0.75	1.03 [0.99–1.06]	0.11	1.00 [0.98–1.04]	0.33
Street canyon effect	Tertile 2 vs 1	1.00 [0.66–1.49]	0.85	0.88 [0.50–1.54]	0.66	1.31 [0.83–2.07]	0.24	0.88 [0.58–1.35]	0.98
	Tertile 3 vs 1	1.15 [0.77–1.71]	0.23	1.17 [0.71–1.94]	0.54	1.31 [0.81–2.10]	0.27	0.91 [0.62–1.74]	0.35
	1 unit increase	1.15 [0.78–1.69]	0.22	1.03 [0.64–1.68]	0.88	1.16 [0.78–1.72]	0.46	1.03 [0.74–1.44]	0.84
Street corridor effect [0–2]	>P50 vs < P50	0.91 [0.58–1.43]	0.65	1.12 [0.62–2.00]	0.7	1.18 [0.70–1.98]	0.56	0.88 [0.58–1.35]	0.55
	2 (max) vs < P50	1.02 [0.68–1.52]	0.75	0.88 [0.52–1.49]	0.63	1.31 [0.81–2.12]	0.22	0.91 [0.62–1.74]	0.65
	1 unit increase	1.02 [0.75–1.39]	0.88	0.88 [0.59–1.31]	0.54	1.12 [0.75–1.61]	0.58	0.97 [0.72–1.31]	0.85

Notes: Models adjusted for sex, age, family composition, reported household income, highest household educational level and year

**Table 3:** Multi-exposure model for psychological distress

GHQ-4	Model 1		Model 2		Model 3		Model 4	
Factor	OR (95% IC)	p	OR (95% IC)	p	OR (95% IC)	p	OR (95% IC)	p
**BC** (1 μg/m^3^ increase)	0.86 [0.61–1.22]	0.40	0.97 [0.69–1.38]	0.87	0.95 [0.65–1.39]	0.78	0.96 [0.64–1.43]	0.84
**Veg. coverage-1 km buffer** (25% increase)	0.85 [0.64–1.14]	0.29	1.05 [0.78–1.40]	0.75	1.06 [0.78–1.44]	0.70	1.03 [0.75–1.41]	0.86
**View of green** (10 units increase)	1.03 [0.84–1.25]	0.77	1.03 [0.84–1.26]	0.78	0.95 [0.85–1.28]	0.64	1.06 [0.87–1.30]	0.57
**Density of tree** (25 trees/100 m increase)	1.07 [0.72–1.60]	0.73	1.12 [0.74–1.67]	0.60	1.07 [0.96–1.06]	0.74	1.06 [0.70–1.59]	0.79
**Noise from multi. Source Lden** (1 dB increase)	1.01 [0.98–1.04]	0.50	1.01 [0.98–1.04]	0.46	1.01 [0.98–1.04]	0.52	1.01 [0.98–1.05]	0.35
**Canyon effect** (1 unit increase)	1.20 [0.80–1.80]	0.38	1.29 [0.83–2.01]	0.26	1.42 [0.91–2.23]	0.12	1.32 [0.84–2.06]	0.23
**Corridor effect** (1 unit increase)	0.96 [0.66–1.41]	0.85	1.04 [0.70–1.55]	0.84	1.03 [0.00-1.53]	0.86	1.10 [0.74–1.63]	0.62
**Social support** (Poor vs strong)					2.13 [1.44–3.13]	< 0.001	1.94 [1.29–2.92]	< 0.001
**Physical activity (**Minimal active vs inactive)					1.07 [0.74–1.55]	0.70	1.13 [0.77–1.66]	0.52
Enough active vs inactive					0.48 [0.30–0.79]	< 0.001	0.55 [0.34–0.88]	0.01
**Chronic condition** (Yes vs no)							2.38 [1.67–3.39]	< 0.001

Notes: Models 2,3, 4 were adjusted for sex, age, family composition, reported household income, highest household educational level and year. Model 3 was additionally adjusted for physical activity and social support. Model 4 was additionally adjusted for chronic condition

*PM*_*2.5*_ was the only pollutant for which no significant association was observed with mental health. Ozone (in tertiles and continuous) was negatively associated with *depressive disorders*. A significant linear trend was observed in the association between *BC*, *PM*_*10*_ (in tertiles) and *depressive disorders*.

No significant association was found between any of the mental health outcomes and the following green exposure indicators: *view of green*, *vegetation coverage (600 m buffer)*, *vegetation coverage (1000 m buffer)* and *visible street vegetation coverage*. A significant negative association was found between the *linear tree density* (in categories) and *sleeping disorders*.

No significant association was found between any of the mental health outcomes and noise from multiple sources, street canyon and street corridor effect.

## Multi-exposure models

Tables 4, 5 and 6 display the results of the multi-exposure models for *psychological distress, anxiety disorders, depressive disorders* and *sleeping disorders*.

**Table 4:** Multi-exposure model for anxiety disorders

ANXIETY DISORDERS	Model 1		Model 2		Model 3		Model 4	
Factor	OR (95% IC)	p	OR (95% IC)	p	OR (95% IC)	p	OR (95% IC)	p
**BC** (1 μg/m^3^ increase)	1.07 [0.76–1.51]	0.68	1.09 [0.76–1.57]	0.62	1.16 [0.78–1.71]	0.47	1.16 [0.77–1.74]	0.47
**Veg. coverage-1 km buffer ** (25% increase)	0.77 [0.51–1.79]	0.21	1.04 [0.70–1.55]	0.83	1.07 [0.72–1.61]	0.72	1.00 [0.65–1.56]	0.97
**View of green** (10 units increase)	1.08 [0.85–1.45]	0.55	1.12 [0.87–1.49]	0.81	1.01 [0.88–1.50]	0.99	1.00 [0.89–1.51]	0.82
**Density of tree** (25 trees/100 m increase)	0.83 [0.47–1.46]	0.51	0.91 [0.51–1.63]	0.37	0.77 [0.45–1.30]	0.31	0.76 [0.44–1.26]	0.29
**Noise from multi. Source Lden** (1 dB increase)	1.01 [0.97–1.05]	0.50	1.00 [0.97–1.04]	0.76	1.00 [0.96–1.03]	0.32	1.00 [0.97–1.04]	0.27
**Canyon effect** (1 unit increase)	1.02 [0.61–1.68]	0.94	1.14 [0.66–1.98]	0.63	1.13 [0.61–2.10]	0.70	1.07 [0.58–1.97]	0.82
**Corridor effect** (1 unit increase)	0.91 [0.56–1.46]	0.69	0.99 [0.59–1.69]	0.99	0.98 [0.57–1.67]	0.94	1.01 [0.59–1.74]	0.97
**Social support** (Poor vs strong)					5.52 [3.57–8.54]	< 0.001	4.99 [3.22–7.74]	< 0.001
**Physical activity (**Minimal active vs inactive)					0.59 [0.35–1.01]	0.06	0.63 [0.37–1.07]	0.09
Enough active vs inactive					0.57 [0.30–1.07]	0.08	0.67 [0.37–1.22]	0.19
**Chronic condition** (Yes vs no)							3.19 [1.89–5.41]	< 0.001

Notes: Models 2,3, 4 were adjusted for sex, age, family composition, reported household income, highest household educational level and year. Model 3 was additionally adjusted for physical activity and social support. Model 4 was additionally adjusted for chronic condition

**Table 5:** Multi-exposure model for depressive disorders

DEPRESSIVE DISORDERS	Model 1		Model 2		Model 3		Model 4	
Factor	OR (95% IC)	p	OR (95% IC)	p	OR (95% IC)	p	OR (95% IC)	p
**BC** (1 μg/m^3^ increase)	1.18 [0.88–1.58]	0.28	2.48 [1.33–4.63]	0.004	2.44 [1.28–4.62]	0.007	2.46 [1.30–4.66]	0.006
**BC (quadratic term)**			0.73 [0.53–0.99]	0.04	0.74 [0.53–1.01]	0.06	0.75 [0.53–1.03]	0.08
**Veg. coverage-1 km buffer** (25% increase)	0.83 [0.61–1.12]	0.24	1.41 [0.96–2.05]	0.08	1.43 [0.96–2.12]	0.08	1.40 [0.92–2.13]	0.11
**View of green** (10 units increase)	1.17 [0.55–1.49]	0.08	1.17 [0.93–1.49]	0.19	1.21 [0.95–1.54]	0.27	1.23 [0.97–1.58]	0.12
**Density of tree** (25 trees/100 m increase)	1.08 [0.64–1.82]	0.14	1.10 [0.62–1.96]	0.18	0.99 [0.57–1.69]	0.13	0.99 [0.81–1.23]	0.08
**Noise from multi. Source Lden** (1 dB increase)	1.03 [0.99–1.06]	0.77	1.02 [0.99–1.05]	0.73	1.02 [0.99–1.05]	0.96	1.03 [0.99–1.06]	0.99
**Canyon effect** (1 unit increase)	1.10 [0.72–1.69]	0.65	1.12 [0.70–1.81]	0.52	1.28 [0.78–2.09]	0.32	1.23 [0.75–2.02]	0.40
**Corridor effect** (1 unit increase)	1.26 [0.79–2.00]	0.31	1.47 [0.88–2.48]	0.14	1.45 [0.87–2.42]	0.16	1.61 [0.96–2.69]	0.07
**Social support** (Poor vs strong)					4.06 [2.74–6.03]	< 0.001	3.82 [2.51–5.80]	< 0.001
**Physical activity** (Minimal active vs inactive)					0.78 [0.52–1.19]	0.25	0.85 [0.55–1.31]	0.46
Enough active vs inactive					0.40 [0.22–0.70]	0.002	0.47 [0.27–0.84]	0.01
**Chronic condition** (Yes vs no)							3.06 [2.06–4.54]	< 0.001

Notes: Models 2,3, 4 were adjusted for sex, age, family composition, reported household income, highest household educational level and year. Model 3 was additionally adjusted for physical activity and social support. Model 4 was additionally adjusted for chronic condition

**Table 6:** Multi-exposure model for sleeping disorders

SLEEPING DISORDERS	Model 1		Model 2		Model 3		Model 4	
Factor	OR (95% IC)	p	OR (95% IC)	p	OR (95% IC)	p	OR (95% IC)	p
**BC (1 μg/m**^3^ increase)	0.98 [0.74–1.29]	0.87	1.21 [0.89–1.64]	0.23	1.22 [0.90–1.66]	0.19	1.26 [0.92–1.71]	0.14
**Veg. coverage-1 km buffer** (25% increase)	0.96 [0.74–1.26]	0.78	1.15 [0.87–1.49]	0.33	1.15 [0.87–1.52]	0.32	1.13 [0.85–1.51]	0.39
**View of green** (10 units increase)	0.97 [0.83–1.10]	0.50	0.97 [0.82–1.01]	0.38	0.97 [0.82–1.15]	0.46	0.98 [0.82–1.16]	0.32
**Density of tree** (25 trees/100 m increase)	0.96 [0.64–1.44]	0.71	0.96 [0.62–1.47]	0.72	0.90 [0.60–1.35]	0.73	0.89 [0.60–1.33]	0.78
**Noise from multi. Source Lden** (1 dB increase)	1.01 [0.98–1.04]	0.86	1.01 [0.98–1.04]	0.85	1.01 [0.98–1.04]	0.61	1.01 [0.99–1.04]	0.58
**Canyon effect** (1 unit increase)	1.03 [0.70–1.51]	0.89	1.05 [0.71–1.56]	0.79	1.03 [0.69–1.53]	0.89	0.96 [0.65–1.44]	0.86
**Corridor effect** (1 unit increase)	0.92 [0.65–1.30]	0.64	0.99 [0.69–1.42]	0.95	0.99 [0.68–1.42]	0.94	1.05 [0.73–1.51]	0.81
**Social support** (Poor vs strong)					2.76 [1.95–3.89]	< 0.001	2.60 [1.84–3.67]	< 0.001
**Physical activity (**Minimal active vs inactive)					0.84 [0.59–1.20]	0.34	0.88 [0.62–1.26]	0.49
Enough active vs inactive					0.91 [0.60–1.37]	0.64	1.00 [0.67–1.51]	0.99
**Chronic condition** (Yes vs no)							2.10 [1.52–2.91]	< 0.001

Notes: Models 2,3, 4 were adjusted for sex, age, family composition, reported household income, highest household educational level and year. Model 3 was additionally adjusted for physical activity and social support. Model 4 was additionally adjusted for chronic condition

In Model 1, which included *BC*, *linear tree density*, *view of green*, *vegetation coverage (1000 m buffer), noise from multiple source (Lden), street corridor effect* and *street canyon effect*, a positive and significant association was observed between *BC* exposure and *depressive disorders.*

In Model 2, which was additionally adjusted for socio-economic factors, the association between *BC* exposure and *depressive disorders* remained. For *depressive disorders*, the quadratic term of *BC* was included in the model since it significantly improved the model. The negative sign of the quadratic term means that the relationship between *BC* and *depressive disorders* is concave instead of linear.

In Model 3, which was additionally adjusted for *physical activity* and perceived *social support*, *BC* exposure remained significantly associated with *depressive disorders.* For *depressive disorders*, the quadratic term of *BC* was included in the model since it significantly improved the model. Poor *social support* was significantly correlated with all mental health outcomes. The *level of physical activity* was also significantly associated with all mental health outcomes, except for *sleeping disorders* [22].

**Fig. 4:**
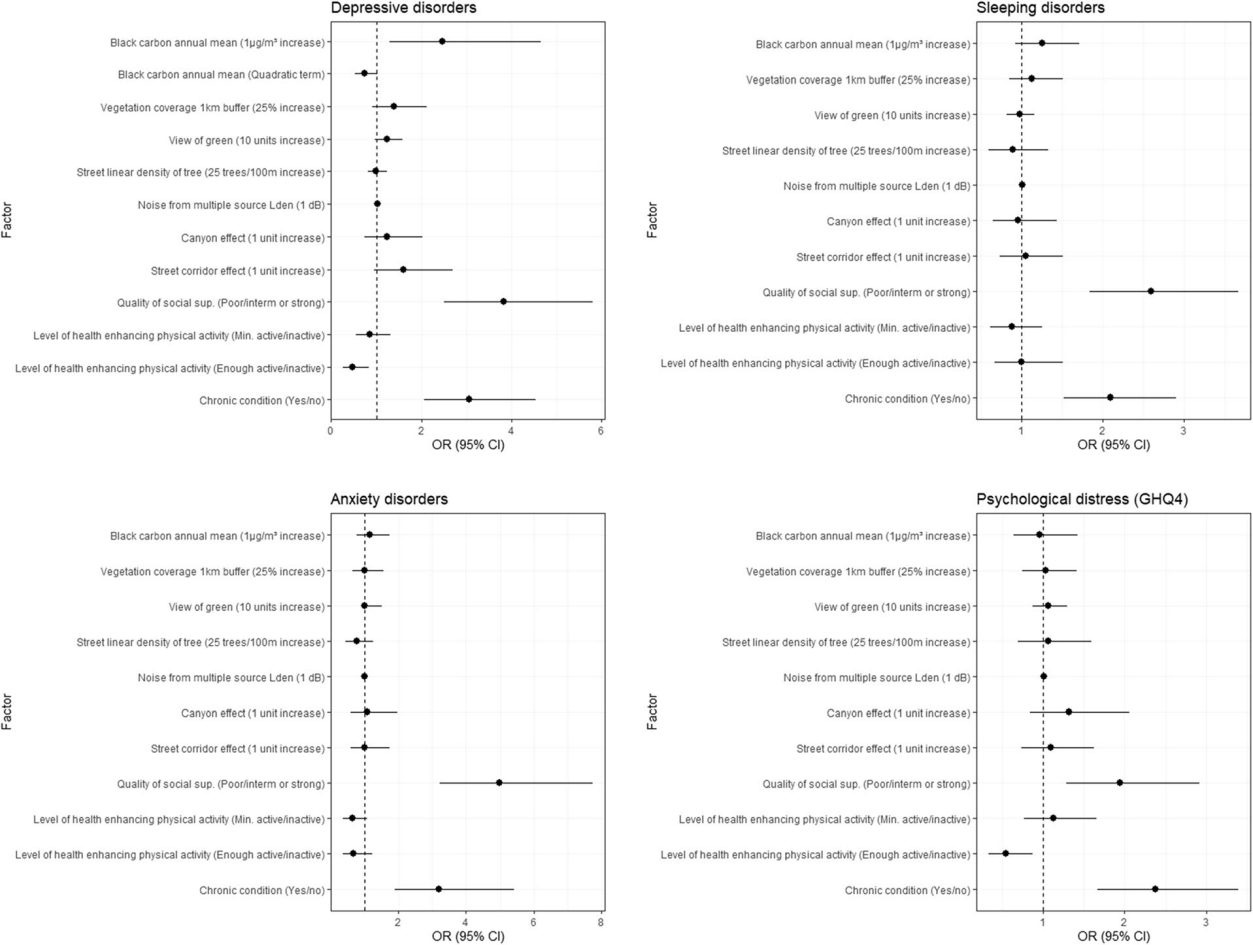
Forest plots of the fully adjusted models (model 4) for each mental health outcome

In Model 4, which was additionally adjusted for *chronic condition*, *BC* exposure remained positively and significantly associated with *depressive disorders* (Fig. 4). For *depressive disorders*, the quadratic term of *BC* was included in the model since it significantly improved the model. The presence of a *chronic condition* was strongly correlated with all mental health outcomes. *Physical activity* and *social support* remained associated with all mental health outcomes, except for *sleeping disorders*.

The mean VIF for model 1, 2, 3 and 4 were respectively 1.6, 1.44, 1.39, and 1.38. None of the VIF exceeded the value of 3.

No significant interactions were found between environmental factors and sex and age.

The Benjamini-Hoshberg method was used to control for multiple hypotheses testing. By taking into account the 41 tests by outcome (41 * 4) and a False Discovery Rate among the significant results of 10%, all the associations between *BC* and *depressive disorders* remained significant.

Forest plots for the other models are available in the additional files: model 1 (Additional file 2), model 2 (Additional file 3), model 3 (Additional file 4).

## Mediation analysis

Both SEMs provided support for the hypothesized inverse association between air pollution and green space (standardized coefficient β_std_ = − 0.61, *p* = 0.02), for the inverse associations between physical activity and distress (β_std_ = − 0.11, *p* < 0.001), and between SES and distress (β_std_ = − 0.19, *p* < 0.001), and for the association between poor social support and distress (β_std_ = 0.32, *p* < 0.001), with an effect of year (*p* = 0.04).

The SEMs did not support our hypothesized direct and indirect associations between green space, air pollution, noise and distress. Indirect effects, calculated by multiplying the coefficient of the unstandardized parameter estimates of the constituent paths are displayed in the additional files (Additional file 6).

**Fig. 5:**
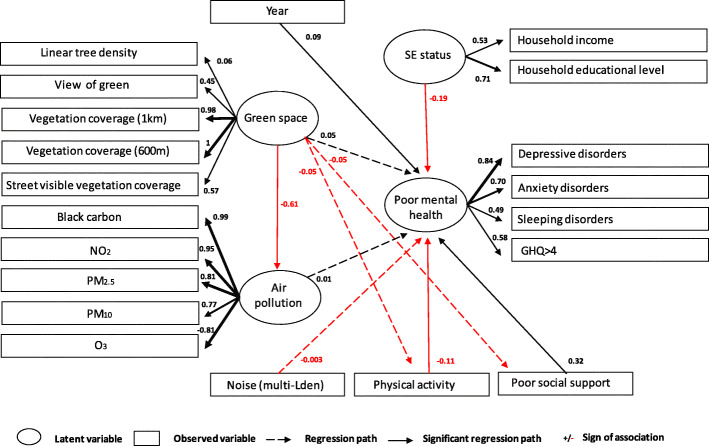
Structural equation model of the associations between green space, air pollution, noise, socioeconomic status (SE status), social support, physical activity and mental health in adults in Brussels (Model 1)

The fit indices pointed toward suboptimal model fit: *p* < 0.001, CFI = 0.68 (threshold> 0.90), RMSEA = 0.172 [95% CI: 0.168–0.175] (threshold< 0.08), SRMR = 0.10 (threshold< 0.08). The chi-square for model 1 and 2 were respectively 6455.868 (df = 161) and 6459.926 (df = 161) and *P* < 0.001 (threshold> 0.05). All coefficients of the SEMs are available in the additional files (Additional file 5). The path diagram of model 1 was visualized in Fig. 5 and the path diagram of model 2 is available in the additional files (Additional file 7).

## Discussion

### Main findings

Our results demonstrate that, in the Brussel-Capital Region, exposure to air pollution was positively associated with *depressive disorders*. *Linear tree density* was negatively associated with *sleeping disorders*, while *vegetation coverage (1000 m buffer), view of green*, *street visible vegetation coverage* and *street canyon effect* were not associated with any of the mental health indicators in this study.

The association between *black carbon* and *depressive disorders* remained significant in multi-exposure models, fully adjusted for lifestyle factors. However, the association between *linear tree density* and *sleeping disorders* was no longer significant in multi-exposure models.

## Comparison with previous studies and potential hypothesis

### Air pollution

To our knowledge, no previous studies evaluated whether *black carbon* exposure was spatially associated with adverse mental health outcomes. In most epidemiological studies, air pollution was mainly approached through *NO*_*2*_ or *PM* exposure. However, it has been shown that *black carbon* represents one of the most health-relevant components of *PM* and could be a valuable indicator to assess the health effects of air quality dominated by primary combustion particles [22, 71].

Although we could not demonstrate an association between *PM*_*2.5*_ exposure and mental disorders, the associations between *BC*, *PM*_*10*_*, NO*_*2*_ and *depressive disorders* suggest a deleterious effect of traffic-related air pollution on mental health. Our observed associations between air pollution and depressive disorders are in line with several previous studies [10, 11, 72–76]. However, results from epidemiological studies on the effect of air pollution on depressive symptoms remain ambiguous. In European cohorts, inconsistent associations have been found between long-term exposure to air pollution and depression but results suggested nevertheless a potentially detrimental relationship [12, 77]. In a recent meta-analysis, Fan et al., confirmed an association between short-term exposure to *NO*_*2*_ and depression, but not with long-term exposure to others pollutants [78]. However, three previous systematic reviews support the hypothesis of an association between long-term exposure to *PM*_*2.5*_ and depression [14, 20, 79]. Despite all those analyses involving large datasets, the available evidence for a causal relationship between air pollution and mental disease is still lacking, on account of a modest effect magnitude and weak support for temporality, biological gradient, coherence and analogy [80].

The observed positive association between air pollution and *depressive disorders* was not attenuated in multi-exposure models fully adjusted for SES and lifestyle factors. *Social support* and *physical activity* were always significantly associated with nearly all mental health outcomes. However, due to the cross-sectional design of the study, we are not able to know whether those factors are causes or consequences of the mental health disorders.

### Urban greenness

Our analysis did not bring out the expected benefits of green exposure on mental health. Our results differ from many studies published recently detailing that higher levels of greenness exposure may lower levels of depressive symptoms [31]. However, contrarily to individual studies recent systematic reviews found limited evidence of mental health benefits from residential greenness [32, 81]. Methodological problems such as the heterogeneity in green exposure assessment, the failure to account for important potential confounders such as air pollution, noise or quality of green space have already been pointed out [32, 81]. In our study, several reasons could explain the fact that an association between green exposure and mental health was not found. Despite our attempt to use vegetation data at different levels (residence, street and neighborhood) and of various sources, it is possible that other characteristics of public and private green spaces may give better results. It has been shown that the strength of the association between green exposure and mental health outcome could differ depending on the green assessment measure [82]. Secondly, the type and the quality of the urban green space was not taken into account in our analysis because this data was not available. Yet biodiversity, accessibility, and social safety are important factors that could influence the use of green spaces and might drive health outcomes. Several studies show that, from a mental health perspective, the quality of public green spaces within a neighborhood could play a more important role compared to quantity [83–85]. Although we can quantify the vegetation coverage in a defined buffer, we cannot assume that everybody has equal access to it. In the BCR, nearly two thirds of the dwellers has no access to high quality green spaces and this mainly concerns the poorest population [86]. This high unequal distribution of green space might be a factor that requires further research, especially because it has been shown that health benefits of access to green space may depend on socioeconomic status [87, 88]. In the BCR, the benefits of urban green spaces do not accrue equally to everybody because the use of green space depends on complex interdependencies between socio-economic characteristics, residential location and attitude towards green space of residents [52]. In the same vein, a recent qualitative research carried out in the BCR (which is also part of the NAMED project) showed that influences of the urban neighborhood environment are rather complex due to a broad range of personal, social, physical, and institutional factors and a constant interplay between those factors [89].

Finally, a last hypothesis is related to selective migration based on people’s health. Depressed people or people with a chronic condition might choose to live in greener environments, which could potentially lead to underestimation of the effects of greenness on depressive disorders.

### Noise

Unlike other previous studies [37], we could not demonstrate a significant association between noise from multiple source (Lden) and mental health. The non-significant findings could be attributed to the fact that we control for too many potential confounders that may lead to an underestimation of the effect [36, 90].

Another potential reason is that mental health was investigated as a direct effect of noise exposure, without taking into account the contextual model in which the effects of noise are realized [91]. The pathway from noise to mental disorders is complex and involves, inter alia, genetic and social factors [92]. It has been shown that the multiple mutual associations between noise, noise sensitivity, sleeping disorders, and mental health disorders represent an important challenge to detect direct associations between noise and mental health [36]. Finally, our models relied on static noise exposure assessment that do not take into account all the environmental influences occurring along the daily movements [93].

## Strengths and weaknesses

We studied several aspects of mental health that until now have mainly been studied separately: *anxiety*, *sleeping* and *depressive disorders* and the presence of *probable mental disorders* (according to the GHQ-4) were assessed through validated questionnaires. We evaluated associations of multiples urban exposures on mental health. Surrounding greenness was approached at different scales, in order to capture a variety of spatial characteristics of the residential environment. Furthermore, this is the first epidemiological study to assess the potential association of *black carbon* and the *street corridor* and *canyon effect* with mental health. Environmental exposure was assessed at the place of residence of participants through objective measures using GIS. To reduce the risk of exposure misclassification, only people living at the same place of residence for more than one year were included in the study.

Our study encountered the common limitations of cross-sectional studies, such as the inability to identify the temporality of the exposure-response relationship. Despite the strong association found between air pollution and *depressive disorders*, the cross-sectional design does not allow to prove a causal effect. Further studies with prospective designs are warranted to identify potential causal associations between air pollution and poor mental health. An important limitation of our study is the potential effect of multiple testing. Due to the high number of tests performed on the same dataset, we cannot exclude a false positive finding. However, many statisticians feel that adding a correction for the Type 1 error (alpha error) for multiple comparisons also increases the chances of a Type 2 error and emphasize the importance of assessing the actual effect size instead of focusing only on statistical significance [94, 95]. Thus, consequences of both type of errors should to be considered, especially if the sample size is small. We used the Benjamini-Hoshberg method to control for multiple hypotheses testing and all the associations between *BC* and *depressive disorders* remain significant.

In our results, apart from the *p*-value, there is still evidence regarding the direction of the odds ratio showing a positive association between *BC* exposure and *depressive disorders*.

Another limitation of our study is the potential exposure misclassification related to the use of residential greenness. It would be helpful to have data not only on the availability of residential green space, but also on the actual use of green spaces, as well as their quality and accessibility. Personal mobility should also be integrated in dynamic exposure assessments to reduce exposure misclassification [93]. The mental health status misclassification cannot be excluded since the assessment of the outcomes was not confirmed by health professionals.

Our study’s analytical plan based on multi-exposure models has some limitations due to the treatment of potential overlapping and correlated exposure as independent variables. Our multi-exposure models with an increasing level of adjustment do not reflect the multiple intertwined pathways working together in the association between urban environment and mental health [96]. A SEM was carried out to analyze this problem and explore the potential mediation effect of air pollution, physical activity and social support in the association between greenness and mental health. Results of the SEM did not support the observed association between air pollution and *depressive disorders* in the regression analysis. This is probably due to the use of a latent variable for mental health and this emphasizes the importance to analyze the different dimensions of mental health separately. Studies based on multi-exposure models usually calculate joint effect measures, based on a cumulative risk index of different environmental factors [97]. In our study, we were not able to calculate them because of the small size of the sample. It has been previously shown that the effect of combined exposure to air pollution and decreased surrounding green on mental health were larger compare to non-cumulative effects [97].

Blue space, an urban design term for visible water, is an important dimension of the urban environment that we did not include in our research. Although studies on blue space and mental health are scarce, there is evidence suggesting that living near blue spaces may have positive effects on the wellbeing [98]. To take into account the socio-economic status of the participants in our analysis, our models were adjusted for the reported household income, but not for the socioeconomic status of the municipalities. However, it is important to mention that the relationship between income and mental health cannot only be explained by the “absolute income hypothesis”, which states that higher individual income improves individual health as more resources can be devoted to health related services or goods. It has been shown that the relative income or the relative deprivation also play an important role, with the most deprived are the most likely to experience frustration and to report a poor mental health [99]. This relative income is an important dimension, especially in the BCR, where there are huge disparities within the municipalities.

Finally, we were limited to the information that was provided in the Health Interview Survey. We performed the analysis on the participants that answered all questions, and did not make inferences on missing data.

## Conclusions

We investigated whether specific features of the urban environment, like air pollution, urban greenness, noise and urban building morphology, such as the *street corridor effect* and the *street canyon effect*, were correlated with different dimensions of mental health of the citizens of the Brussels-Capital Region. Our results suggest that traffic-related air pollution (*black carbon, NO*_*2*_*, PM*_*10*_) exposure was positively associated with higher odds of *depressive disorders*. No association between urban greenness, noise, building morphology and mental health could be demonstrated. Strong effect size was found for air pollution and *depressive disorders*: after controlling for all covariates, the odds of *depressive disorders* were twice as high for those who were highly exposed to *black carbon* compared to those who were less exposed. This association remained in multi-exposure models adjusted for greenness, noise, urban morphology and socio-economic factors.

These findings have important implications because the majority of the BCR population resides in areas where particulate matter concentrations are above the WHO guidelines. This suggests that public policies aiming at reducing traffic related-air pollution may play a role in also reducing the burden of depressive disorders in the Region of Brussels.

## Supplementary Information

**Additional file 1.** Spearman correlation matrix of the environmental factors.

**Additional file 2.** Forest plots of the fully adjusted models (model 1) for each mental health outcome.

**Additional file 3.** Forest plots of the fully adjusted models (model 2) for each mental health outcome.

**Additional file 4.** Forest plots of the fully adjusted models (model 3) for each mental health outcome.

**Additional file 5.** Results of the SEM mediation analysis.

**Additional file 6.** Unstandardized estimates of indirect effect of green space on mental health calculated in model 1 and model 2.

**Additional file 7.** Structural equation model of the associations between green space, air pollution, noise, socioeconomic status (SE status), social support, physical activity and mental health in adults in Brussels (Model 2).

## Data Availability

All the references cited in the manuscript are included in the Reference list. Data sets analyzed during the current study are not publicly available due to protection of personal data. More information on the Health Interview Survey, as well as enquiries for access to this data, can be found here: https://his.wiv-isp.be/SitePages/Home.aspx.

## Abbreviations

BC: Black carbon
BCR: Brussels-Capital Region
GIS: Geographical Information System
GHQ: General Health Questionnaire
HIS: Health Interview Survey
IPAQ: International Physical Activity Questionnaire
IQR: Inter Quartile Range
MEHM: Minimum European Health Module
NAMED: Nature Impact on Mental Health Distribution
NDVI: Normalized Difference Vegetation Index
PCA: Principal Component Analysis
PM: Particulate matter
SES: Socio-economic status
SEM: Structural equation modelling
VIF: Variance Inflation Factor
WHO: World Health Organization
