# Supplementary figures and images for: Association between urban environment and mental health in Brussels, Belgium
Source: BMC Public Health. 2021 Apr 1;21:635. doi: 10.1186/s12889-021-10557-7 (PMC8015067; doi:10.1186/s12889-021-10557-7)

Spearman correlation matrix

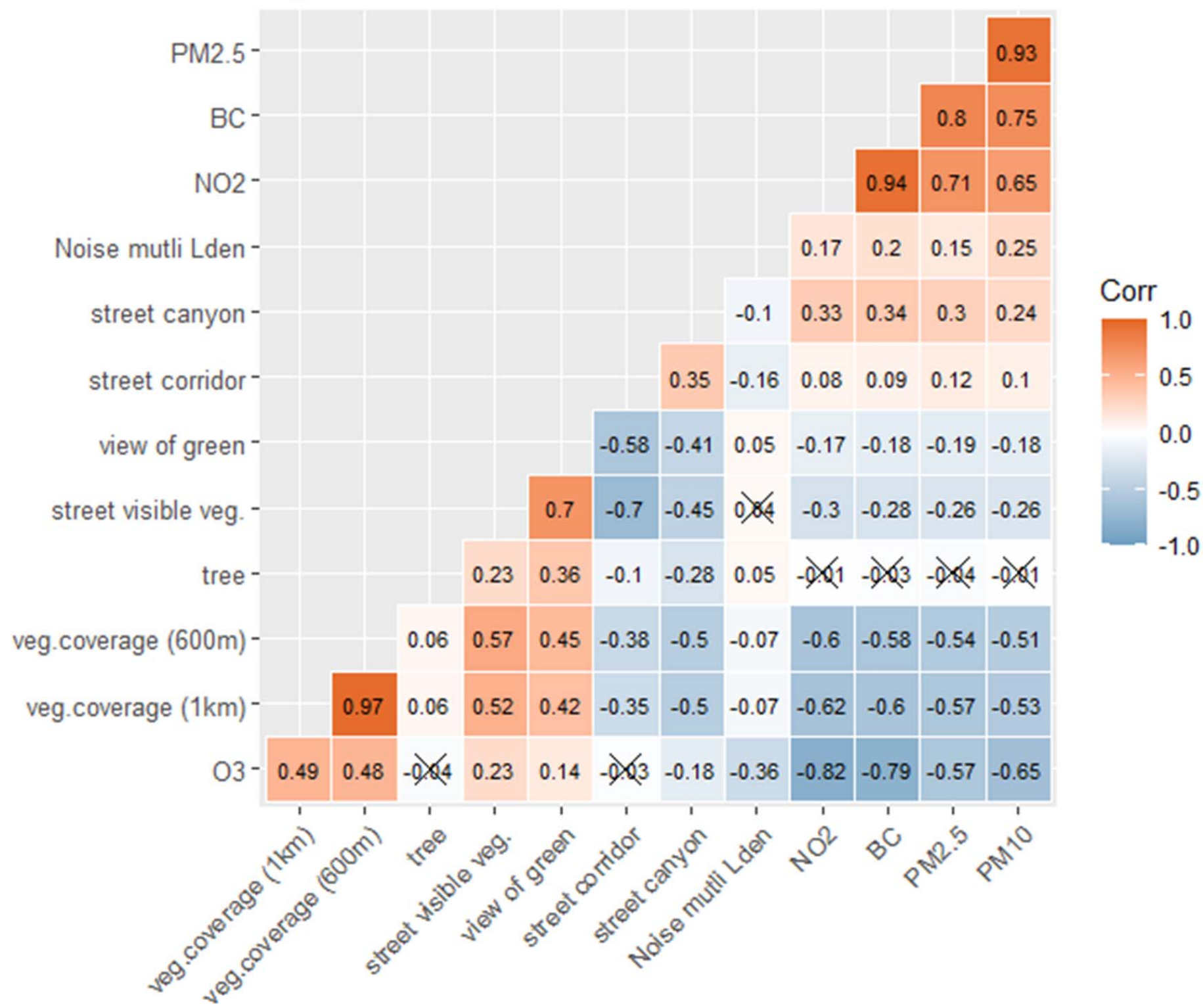

Supplement: Supplementary file 1 — Additional file 1. Spearman correlation matrix of the environmental factors. [file 12889_2021_10557_MOESM1_ESM.pdf]

Depressive disorders

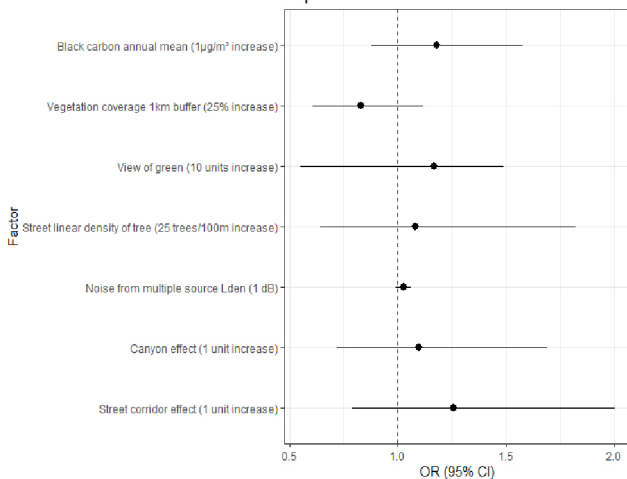

Sleeping disorders

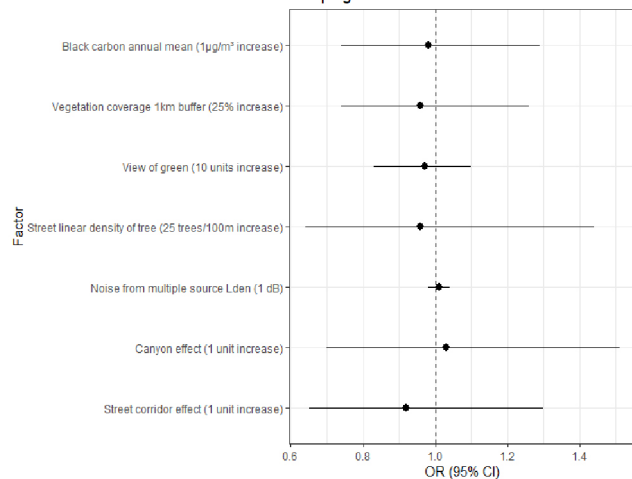

Anxiety disorders

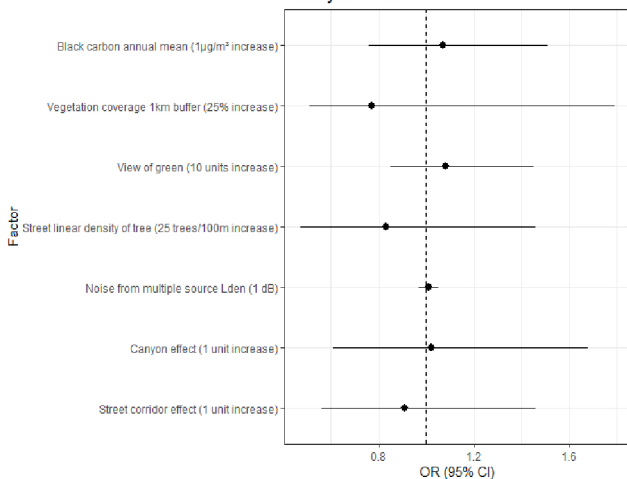

Psychological distress (GHQ4)

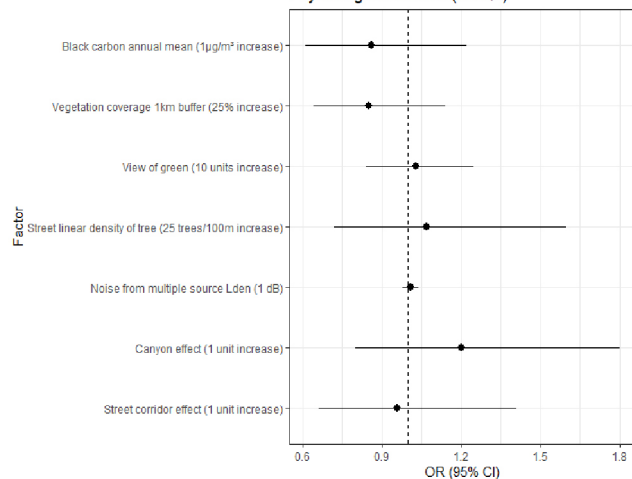

Supplement: Supplementary file 2 — Additional file 2. Forest plots of the fully adjusted models (model 1) for each mental health outcome. [file 12889_2021_10557_MOESM2_ESM.pdf]

Depressive disorders

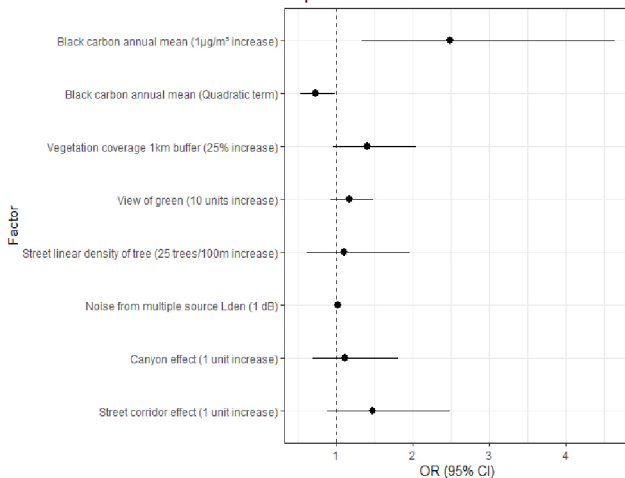

Sleeping disorders

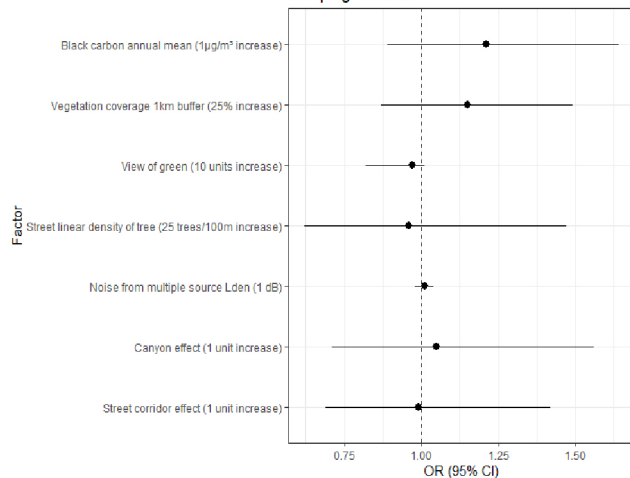

Anxiety disorders

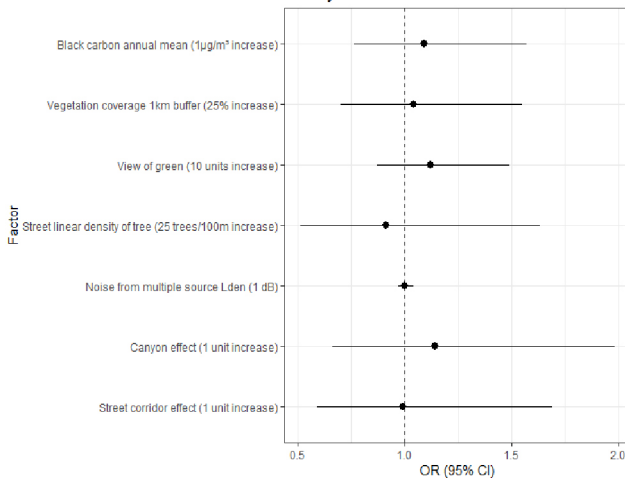

Psychological distress (GHQ4)

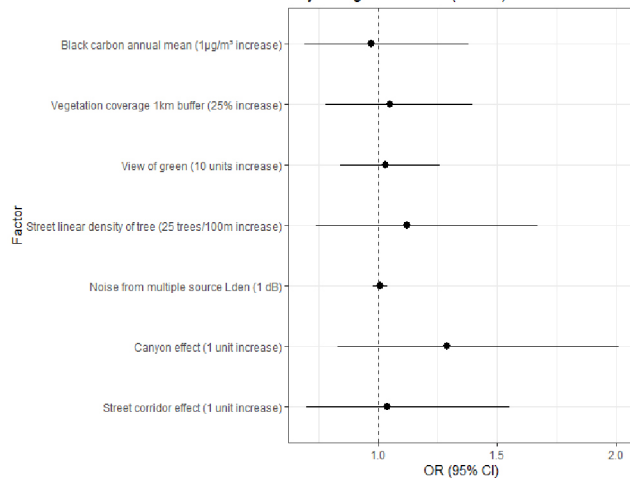

Supplement: Supplementary file 3 — Additional file 3. Forest plots of the fully adjusted models (model 2) for each mental health outcome. [file 12889_2021_10557_MOESM3_ESM.pdf]

Depressive disorders

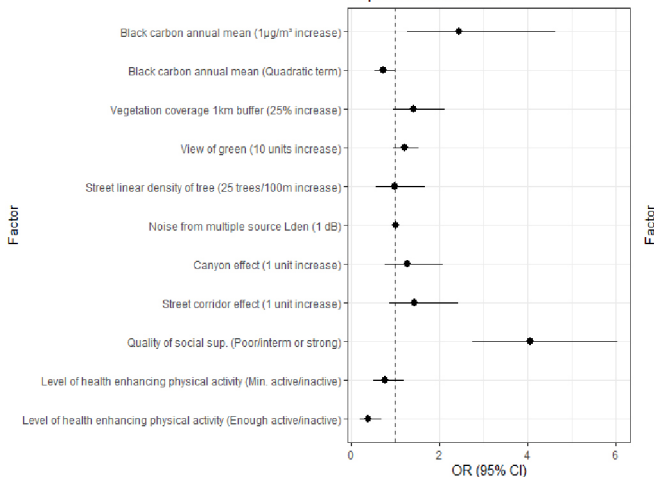

Sleeping disorders

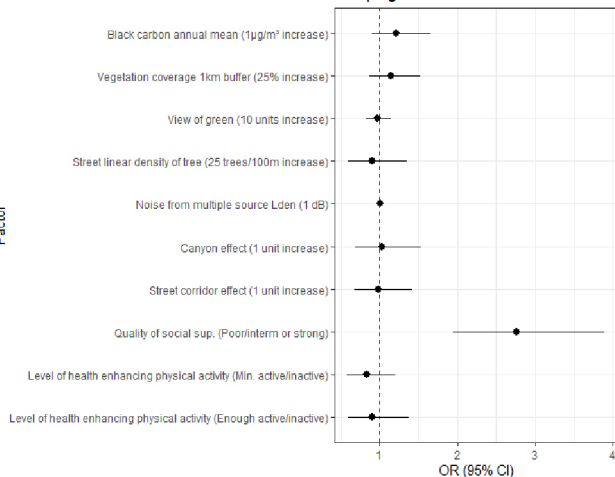

Anxiety disorders

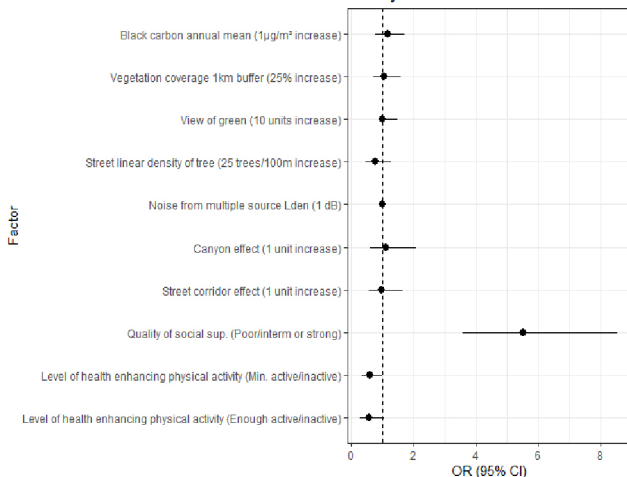

Psychological distress (GHQ4)

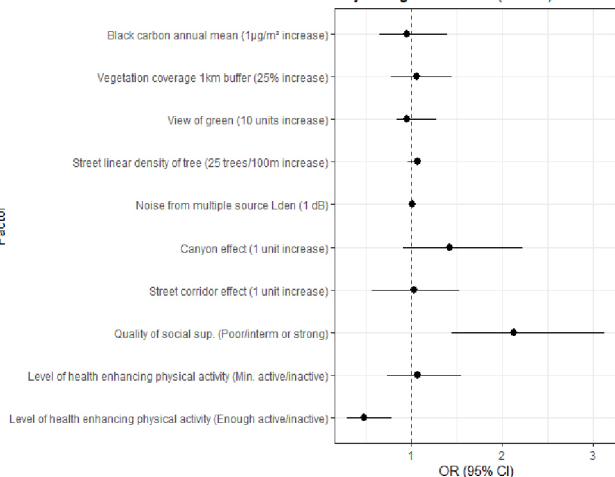

Supplement: Supplementary file 4 — Additional file 4. Forest plots of the fully adjusted models (model 3) for each mental health outcome. [file 12889_2021_10557_MOESM4_ESM.pdf]

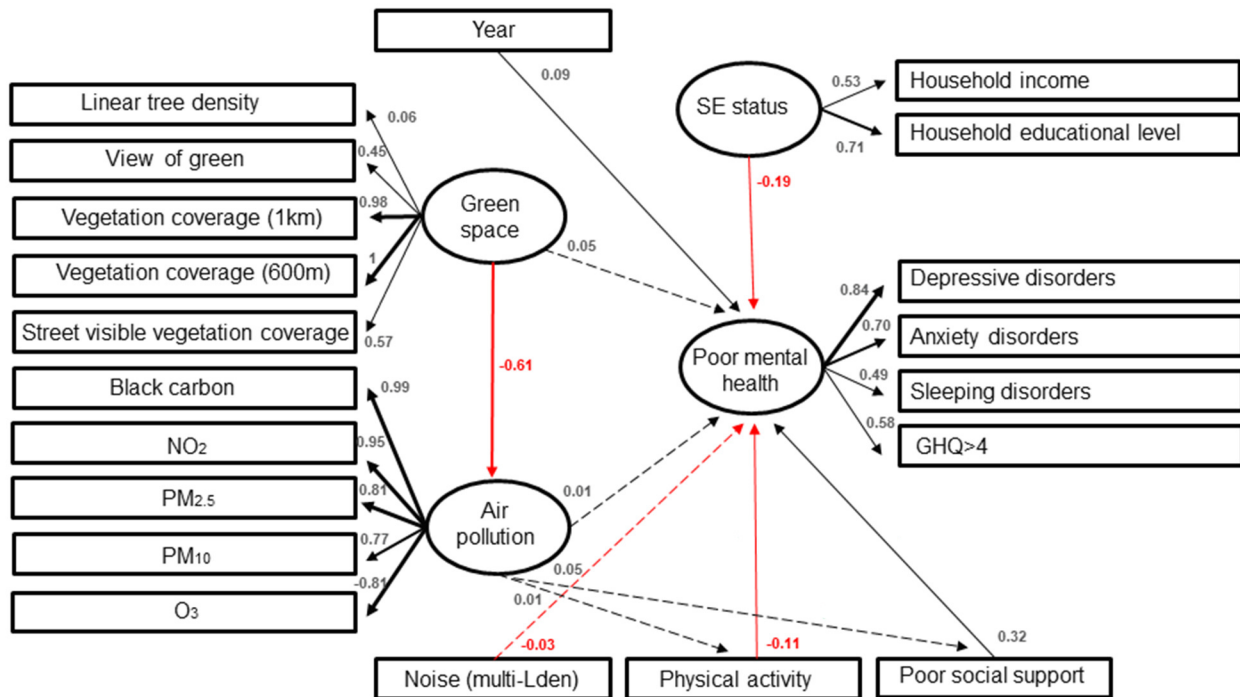

Supplement: Supplementary file 7 — Additional file 7. Structural equation model of the associations between green space, air pollution, noise, socioeconomic status (SE status), social support, physical activity and mental health in adults in Brussels (Model 2). [file 12889_2021_10557_MOESM7_ESM.pdf]
