# Supplementary material for: Association between urban environment and mental health in Brussels, Belgium
Source: BMC Public Health. 2021 Apr 1;21:635. doi: 10.1186/s12889-021-10557-7 (PMC8015067; doi:10.1186/s12889-021-10557-7)
Supplement: Supplementary file 5 — Additional file 5. Results of the SEM mediation analysis. [file 12889_2021_10557_MOESM5_ESM.pdf]

Latent Variables	Estimate	SE	z	p	Std.all
Mental health					
Depressive disorders	1.000				0.840
Anxiety disorders	0.660	0.032	21.850	<0.001	0.700
Sleeping disorders	0.690	0.040	16.060	<0.001	0.490
GHQ-4	0.740	0.044	18.900	<0.001	0.580
Green space					
Linear tree density	1.000				0.062
View of green	7.142	3.140	2.242	0.025	0.451
Vegetation coverage (1km)	15.491	6.769	2.259	0.024	0.976
Vegetation coverage (600m)	16.776	6.893	2.259	0.024	0.996
Street visible vegetation coverage (and 10 meters on either side)	8.977	3.948	2.250	0.024	0.569
Air pollution					
Black carbon	1.000				0.988
NO2	0.959	0.011	90.368	<0.001	0.948
PM2.5	0.820	0.017	48.038	<0.001	0.811
PM10	0.776	0.019	41.822	<0.001	0.766
O3	-0.820	0.019	-38.901	<0.001	-0.810
Socio-economic status					
Reported household income	1.000				0.530
Highest household educational level	1.070	0.140	7.350	<0.001	0.710
Noise					
Multi (Lden)	1.000				1.000
**Model 1**					
**Regression**					
Air pollution ~green space	-9.543	4.181	-2.283	0.022	-0.609
Physical activity ~green space	-0.652	0.438	-1.487	0.137	-0.054
Social support ~green space	-0.349	0.236	-1.478	0.139	-0.053
Mental health ~					
Air pollution	0.005	0.012	0.399	0.690	0.015
Physical activity	-0.047	0.012	-4.002	<0.001	-0.115
Social support	0.243	0.022	11.107	<0.001	0.324
Noise	-0.001	0.009	-0.098	0.922	-0.003
Socio-economic status	-0.112	0.025	-4.407	<0.001	-0.191
Year	0.011	0.004	3.138	<0.001	0.090
Green space	0.026	0.191	0.137	0.891	0.005
**Covariance**					
Green space~~					
Socio-economic status	0.010	0.005	2.151	0.031	0.294
Noise	-0.004	0.003	-1.671	0.095	-0.068
Socio-economic status ~~noise	-0.109	0.022	-4.932	<0.001	-0.205
**Model 2**					
**Regression**					
Air pollution ~green space	-9.549	4.186	-2.281	0.023	-0.609
Physical activity ~air pollution	0.008	0.021	0.355	0.723	0.010
Social support~air pollution	0.021	0.012	1.835	0.067	0.051
Mental health ~					
Air pollution	0.005	0.012	0.399	0.690	0.015
Physical activity	-0.047	0.012	-4.007	<0.001	-0.115
Social support	0.243	0.022	11.108	<0.001	0.324
Noise	-0.001	0.009	-0.098	0.922	-0.030
Socio-economic status	-0.112	0.025	-4.407	<0.001	-0.191
Year	0.011	0.004	3.138	<0.001	0.090
Green space	0.026	0.191	0.137	0.891	0.005
**Covariance**					
Green space~~					
Socio-economic status	0.010	0.005	2.150	0.032	0.294
Noise	-0.004	0.003	-1.670	0.095	-0.068
Socio-economic status ~~noise	-0.109	0.022	-4.932	<0.001	-0.205
