# Supplementary material for: Association between urban environment and mental health in Brussels, Belgium
Source: BMC Public Health. 2021 Apr 1;21:635. doi: 10.1186/s12889-021-10557-7 (PMC8015067; doi:10.1186/s12889-021-10557-7)
Supplement: Supplementary file 6 — Additional file 6. Unstandardized estimates of indirect effect of green space on mental health calculated in model 1 and model 2. [file 12889_2021_10557_MOESM6_ESM.pdf]

Model 1	Estimate	SE	z	p
Indirect effect through air pollution	-0.044	0.112	-0.393	0.690
Indirect effect through physical activity	0.03	0.022	1.394	0.160
Indirect effect through social support	-0.085	0.058	-1.465	0.143
Indirect effect through air pollution, physical activity and social support	-0.098	0.127	-0.772	0.440
Model 2				
Indirect effect through air pollution and subsequently physical activity	0.003	0.01	0.349	0.720
Indirect effect through air pollution and subsequently social support	-0.049	0.03	-1.418	0.156
Indirect effect through air pollution and subsequently physical activity and social support	-0.046	0.03	-1.305	0.192
